# Diverging temperature responses of CO_2_ assimilation and plant development explain the overall effect of temperature on biomass accumulation in wheat leaves and grains

**DOI:** 10.1093/aobpla/plw092

**Published:** 2017-01-09

**Authors:** Iman Lohraseb, Nicholas C. Collins, Boris Parent

**Affiliations:** 1 Australian Centre for Plant Functional Genomics, University of Adelaide, School of Agriculture Food and Wine, Hartley Grove, Urrbrae, South Australia, 5064, Australia; 2 Present address: INRA, UMR759 Laboratoire d’Ecophysiologie des Plantes sous Stress Environnementaux. Place Viala, F-34060 Montpellier, France

**Keywords:** Biomass, development, grain growth, photosynthesis, respiration, specific leaf area, temperature, thermal time, wheat

## Abstract

There is a growing consensus in the literature that rising temperatures influence the rates of biomass accumulation by shortening the development of plant organs and the whole plant and by altering the rates of respiration and photosynthesis. A model describing the net effects of these processes on biomass would be useful, but would need to reconcile reported differences in the effects of night and day temperature on plant productivity. In this study, the working hypothesis was that the temperature responses of CO_2_ assimilation and plant development rates were divergent, and that their net effects could explain observed differences in biomass accumulation. In wheat (*Triticum aestivum*) plants, we followed the temperature responses of photosynthesis, respiration and leaf elongation, and confirmed that their responses diverged. We measured the amount of carbon assimilated per ‘unit of plant development’ in each scenario and compared it to the biomass that accumulated in growing leaves and grains. Our results suggested that, up to a temperature optimum, the rate of any developmental process increased with temperature more rapidly than that of CO_2_ assimilation and that this discrepancy, summarised by the CO_2_ assimilation rate per unit of plant development, could explain the observed reductions in biomass accumulation in plant organs under high temperatures. The model described the effects of night and day temperature equally well, and offers a simple framework for describing the effects of temperature on plant growth.

## Teaser

Under rising temperature, the rate of any developmental process increased with temperature more rapidly than that of CO_2_ assimilation. We found that this discrepancy, summarised by the CO_2_ assimilation rate per unit of plant development, could explain the observed reductions in biomass accumulation in leaves and grain under high temperatures. This simple model describes the effects of night and day temperature equally well, and offers a simple framework for describing the effects of temperature on plant growth, without any supplementary effect of rising night temperatures.

## Introduction

High temperatures decrease biomass accumulation in plant leaves (Vile *et al.* 2012), cereal grains (Wheeler *et al.* 1996) and whole plants, with implications for agricultural productivity and ecology under a climate change scenario (Peng *et al.* 2004). An emerging consensus is that carbon balance is a critical factor in responses of biomass accumulation processes to temperature changes. This view comes from studying temperature responses of grain dry mass (Wardlaw 1994; Wheeler *et al.* 1996), and leaf dry mass per area (LMA) or its reciprocal, the specific leaf area (Poorter *et al.* 2009). Most of these studies investigated the effect of very high temperatures within the ‘stressing range’ where photosynthesis was demonstrated to be negatively affected (Loveys *et al.* 2002; Vasseur *et al.* 2011). Accordingly, high CO_2_ or light, which increases photosynthesis, can partially offset the impact of high temperature on biomass accumulation in vegetative tissues (Taub *et al.* 2000; Vasseur *et al.* 2011) and in grains (Wardlaw 1994; Wheeler *et al.* 1996).

By contrast, rising temperatures in the ‘non-stressing’ temperature range increase the rate of photosynthesis (Atkin and Tjoelker 2003; Sage and Kubien 2007). One consequence is accelerated dry weight accumulation in the grain (Wheeler *et al.* 1996), which reflects faster accumulation of photosynthate. High temperatures also accelerate cell expansion and division, and hasten genetic programs of organ differentiation, consequently shortening the period over which biomass can accumulate (Parent *et al.* 2010a). These effects are largely independent of variations in carbon fixation (Morita *et al.* 2005). Temperature during grain filling impacts final single grain weight with effects on both the rate and duration of grain filling (Sofield *et al.* 1977; Yin *et al.* 2009). Similarly, temperature influences LMA by impacting photosynthesis and the rates of leaf expansion (Tardieu *et al.* 1999).

Predicting temperature effects on biomass accumulation requires an understanding of the dynamics of carbon assimilation and plant development responses. The temperature response of respiration and photosynthesis are now well-described under the ‘non-stressing’ temperature range (Atkin and Tjoelker 2003; Sage and Kubien 2007). These responses are divergent (Atkin *et al.* 2007), and both change after exposure to a period of high temperature, *i.e.* they show acclimation behaviour (Atkin *et al.* 2006; Campbell *et al.* 2007). Parent and Tardieu (2012) demonstrated that multiple developmental processes followed a common temperature response curve within a given species. Indeed, rates of processes as diverse as leaf expansion, progression towards flowering or other developmental milestones (*e.g.* percentage of final grain fill duration per day  =  grain development rate), shared similar temperature responses and are hereafter referred to as ‘development rates’. The temperature responses of these developmental processes followed different patterns to photosynthesis, and other enzymatic reactions involved in primary metabolism (Parent *et al.* 2010a).

However, in crop temperature response models, different formalisms are currently used to describe development and leaf expansion (Parent and Tardieu 2014; Kumudini *et al.* 2014). Predicted responses of development to temperature depend on the chosen equation and its parameterisation, and few models consider equations that accommodate different day and night temperature (example: Crop Heat Unit, reviewed by Kumudini *et al.* 2014), or different plant stages. There are currently efforts from the community of crop modellers to make these equations converge (Makowski *et al.* 2015) with suites of tools such as APSIM (Rosenzweig *et al.* 2013). The same applies to the response of photosynthesis or radiation use efficiency, with several equations used in the various models (reviewed in Parent and Tardieu 2014). While many crop models consider specific leaf area to be a result of leaf expansion and biomass, many others consider SLA as a genetic parameter with leaf expansion being driven by leaf biomass (reviewed in Parent and Tardieu 2014). In addition, there is still debate about specific night temperature effects on biomass or production (Peraudeau *et al.* 2015; Fang *et al.* 2015; Glaubitz *et al.* 2014; Kanno and Makino 2010; Peng *et al.* 2004).

Due to the different and non-linear temperature response curves of development rate, photosynthesis, and respiration, the relative impacts of these component traits on biomass accumulation (and their temperature dynamics) would depend on the particular growth temperature range. Here, we address these divergences by using rates of respiration, photosynthesis and various developmental processes observed across a range of thermal scenarios in wheat to model the temperature responses of these traits. We then express the net photoassimilate accumulation per ‘unit of leaf development’ or ‘unit of grain development’ or ‘unit of whole plant development’ at a given temperature in terms of the equivalent value at 20 °C. As such, this approach provides a framework for describing the relative contributions of photosynthesis and respiration to biomass accumulation across a temperature range, with reference to a standard unit.

## Methods

### Plant growth conditions

All experiments were carried out with the bread wheat (*Triticum aestivum*) cultivar Apogee. Seeds were sown in plastic pots (8 × 8 × 20 cm) filled with a coir-peat-based potting mix. Plants were grown in several identical growth chambers (GC-20 Bigfoot series, BioChambers, Winnipeg, Canada). The light was supplied by fluorescent bulbs (Photosynthetically Active Radiation, PAR  =  380 µmol m^−^^2^ s^−^^1^) for 12 h of photoperiod (PP) with an overall daily PAR (3.6 ± 0.1 MJ m^−^^2^ d^−^^1^) similar to that observed in the field at vegetative stage (O'Connell *et al.* 2004). CO_2_ naturally varied during the day but daily average CO_2_ concentration was similar in all treatments. In each of the three experiments, plants were initially grown under temperatures of 25 °C day (T°_day_) and 20 °C night (T°_night_) and the soil was watered close to the saturation level.

In Experiment 1, plants were transferred to different constant temperatures (11, 17, 20, 23 and 29 °C) at the appearance of leaf 6. Leaf temperature, measured with an infrared thermometer (Raynger MX4, Raytek Corporation, Santa Cruz, CA, USA), was close (ΔT° < 1 °C) to the air temperature, during both nights and days. Because air relative humidity was stable in all treatments (60 ± 5 %), vapour pressure deficit varied from 0.5 kPa at 11 °C to 1.8 kPa at 29 °C.

In Experiment 2, plants at the appearance of leaf 4 were transferred to several thermal regimes (T°_day_/T°_night_: 20/15, 20/20, 25/15 and 25/20 °C) where they remained until anthesis (appearance of first anthers on the main spike).

In Experiment 3, plants at anthesis were transferred to several thermal regimes (T°_day_/T°_night_: 20/15, 20/20, 25/15 and 25/20 °C) where they remained until maturity. At heading (head of the main tiller fully emerged), plants were pruned leaving the main tiller with its three youngest leaves. New tillers were then removed weekly.

### Leaf measurements

In Experiments 1 and 2, leaf elongation rate (LER) was measured on leaf 6, by measuring leaf length with a ruler, at leaf appearance and again after a further 24 h. In parallel, it was determined that this developmental stage corresponded to the linear phase of elongation under all tested thermal scenarios (data not shown).

In Experiments 1 and 2, photosynthesis rate during the day and respiration rate during the night were analysed on fully-developed leaf 4 when leaf 6 was elongating, using a gas exchange system (LI-6400, Li-Cor, Lincoln, NE). Photosynthesis was measured at least 2 h after the lights were switched on and 2 h before the lights were switched off. Artificial illumination was supplied from a red-blue LED light source with PAR  =  380 µmol m^−^^2^ s^−^^1^, similar to the growth chambers, or under saturating light (PAR  =  2000 µmol m^−^^2^ s^−^^1^). Respiration rate during the night was measured at predawn, during the last 3 h of the night cycle. CO_2_ was maintained at 400 ppm (Reference) using the CO_2_ mixer (flow rate  =  500 µmol s^−^^1^).

The daily net photosynthesis rate during the day (P_N_, mol m^−^^2^ d^−^^1^) and daily respiration rate during the night (R, mol m^−^^2^ d^−^^1^) were calculated by integrating the measured instantaneous rates of photosynthesis and respiration during the night during the respective times of light and dark (12 h) to arrive at a daily integral. The overall net CO_2_ assimilation rate per day (A_N_, mol m^−^^2^ d^−^^1^) was calculated:

(Eq.1) $$A_{N}=P_{N}-R$$

Unless indicated otherwise, values of A_N_ and P_N_ used were those measured at PAR  =  380 µmol m^−^^2^ s^−^^1^.

In Experiment 2, leaves 4, 5, 6 and 7 were collected at anthesis. Leaf length was measured with a ruler, leaf area was measured with a planimeter (PATON electronic belt driven planimeter, CSIRO, Canberra, Australia) and leaf dry weight was determined after 2 days at 85 °C.

### Data analysis

The R language (R Development Core Team 2005) was used for all statistical analyses and model regressions, namely a comparison of means (function *pairwise.t.test* with ‘BH’ method), Pearson correlation tests (function *cor.test*), linear regression (function *lm)*, non-linear regression (function *nls*) and analysis of variance (function *anova*). Data and scripts are available on demand.

### Temperature responses

Temperature responses were described by the equation of Johnson *et al.* (1942), modified by Parent and Tardieu (2012):

(Eq.2) $$F(T)=\frac{ATe^{\left(\frac{-\Delta H_{A}^{\text{‡}}}{RT}\right)}}{1+{\left[e^{\left(\frac{-\Delta H_{A}^{\text{‡}}}{RT}\right)}\right]}^{\alpha \left(1-\frac{T}{T_{0}}\right)}}$$

where *F(T)* is the considered rate, *T* is the temperature (Kelvin, K), *Δ*$H_{A}^{\text{‡}}$ (J mol^−^^1^) is the enthalpy of activation of the process and determines the curvature at low temperature, *α* (dimensionless) determines how sharp is the decrease in rate at high temperature and is fixed at 3.5 for development processes (Parent and Tardieu 2012), T_0_ (K) determines the temperature at which the rate is maximum, and A is the trait scaling coefficient. Temperature responses of LER, P_N_, and R were calculated by non-linear regressions on the values measured in Experiment 1. The response of A_N_ to temperature was then calculated from the temperature responses of R and P_N_, using Eq.1.

### Thermal compensation of time and rates

For any measured rate *J*(*T*) at temperature *T*, a temperature compensated rate was calculated as the equivalent rate at 20 °C.

(Eq.3) $$J_{20{}^{\circ}C}=J(T)\frac{F\left(20{}^{\circ}C\right)}{F(T)}$$

with *F(T)* being the response of development to temperature (here the response of LER). Because developmental time (or thermal time t_20°C_) is the reciprocal of development rate, it results in:

(Eq.4) $$t_{20{}^{\circ}C}=t(T)\frac{F\left(T\right)}{F(20{}^{\circ}C)}$$

Such a procedure was already applied in different studies of developmental processes (Louarn *et al.* 2010; Parent *et al.* 2009; Parent *et al.* 2010b), and was applied here for biomass accumulation processes and net CO_2_ assimilation rate (A_N_).

In Experiment 2 and 3, $\frac{F\left(20{}^{\circ}C\right)}{F(T)}$ was calculated in each thermal treatment from LER values directly measured in Experiment 2. In the other cases, $\frac{F\left(20{}^{\circ}C\right)}{F(T)}$ was inferred from the regression function *LER(T)*.

### Leaf senescence profiles

In Experiment 3, chlorophyll content was measured with a SPAD chlorophyll meter (Minolta, Plainfield, Illinois, USA). Each measurement was the average of 15 readings: 5 taken from along each of the three last-developed leaves. In each treatment, four plants were measured repeatedly: at anthesis and at 7, 13, 19, 25, 31, 38, 42 and 46 days after anthesis.

In each thermal scenario, a bilinear model was fitted to the dataset (**see Supporting Information—Methods S1**). It comprised a constant value (SPAD_0_) until a time of senescence (t_s_), followed by a linear decrease in content after this point, with a slope a_s_. Because plants had the same thermal treatment before anthesis, SPAD_0_ was fixed for all thermal scenarios and equalled the average value at anthesis for all treatments (SPAD_0 _ = _ _57.3). A similar procedure was carried out considering time *t* and t_s_ as developmental time (*t*_20°C_ and t_s.20°C_, d_20°C_).

### Biomass accumulation in the grain

In Experiment 3, the main spikes of four plants per thermal scenario were collected at 7, 13, 19, 25, 31 days after anthesis and at grain maturity, and seed number and average single grain dry weight (GDW) were measured after three days at 85 °C. Spikes with fewer than 30 seeds were not used in the analysis (6 in total were discarded from the whole experiment; *n* ≥ 3 was used for all sampling dates and thermal treatments).

Curves of biomass accumulation in the grain can be described with a 3 parameter logistic equation (Morita *et al.* 2005), modified here to obtain the theoretical grain weight at anthesis (W_0_, mg) as a parameter of the following equation (**see Supporting Information—Methods S1**):

(Eq.5) $$W\left(t\right)=\frac{W_{0}\left(1+e^{\left(\lambda \;t_{0}\right)}\right)}{1+e^{\left(-\lambda \;\left(t-t_{0}\right)\right)}}$$

*W(t)* is the weight of one seed (mg) at time *t* (in days) after anthesis, λ (in d^−^^1^) is the slope factor controlling the steepness of the curve and *t*_0_ is the inflection point, or time at which the seed is half the final weight. Because the plants were transferred to the different thermal treatments at anthesis, W_0_ was considered as common in all treatments (W_0 _ = _ _1.65 mg, **see Supporting Information—Methods S1**).

Eq.5 was fitted in each thermal scenario, considering either time or developmental time (*t_20°C_* in d_20°C_). In the last case, the two free parameters are expressed with developmental time units (*t*_0.20°C_ in d_20°C_; λ_20°C_ in d_20°C_^−^^1^). Because *t*_0.20°C_ values were similar between treatments, a single *t*_0_20°C_ value common to all treatments was determined (**see Supporting Information—Methods S1**). Respective values of *t*_0_ were then calculated in each treatment. In this case, λ is the only free parameter.

The grain growth rate *GGR(t)*, was obtained by derivation of Eq.5 (**see Supporting Information—Methods S1**). The grain growth rate is maximal (GGR_max_) at the inflection point, namely *t*_0_.

(Eq.6) $${GGR}_{max}=GGR({\;t}_{0})=\frac{{\lambda \;W}_{0}\left(1+e^{\left(\lambda {\;t}_{0}\right)}\right)}{4}$$

with time and model parameters expressed either with time or developmental time units.

Note that with *t*_0.20°C_ and W_0_ fixed, GGR_max.20°C_ depends only on λ_20°C_ (and the reciprocal, λ_20°C_ depends only on GGR_max.20°C_). GGR_max.20°C_ alone can therefore explain the kinetics of grain growth rate.

Grain filling duration (t_f_) was calculated as the duration between anthesis and the time at which the grain reached 95% of its final weight (**see Supporting Information—Methods S1**).

(Eq.7) $$t_{f}=-\frac{1\;}{\lambda }\ln\left[\frac{5\;}{95}\right]+t_{0}$$

### Grain growth simulations

For any thermal scenario, a time series (0 to 100 days after anthesis, time step =1 d) was built, with corresponding photoperiod *PP(t)*, *T°_day_(t)*, *T°_night_(t)* and *T°_ave_(t). t_20°C_(t)*, *P_N_(t)*, *R(t)* were calculated from parameters of Eq.2 (parameter values differing between processes). *A_N.20°C_(t)* was calculated from Eq.1 and 3. *λ _20°C_ (t)* was inferred from the linear relationship between λ _20°C_ and A_N.20°C_ obtained in Experiment 3. *GGR*_20°C_ *(t)* was calculated (**see Supporting Information—Methods S1**) and individual grain weight was then obtained at each *t* by numerically integrating *GGR*_20°C_ between anthesis and the corresponding *t_20°C_(t)*.

(Eq.8) $$W\left(t\right)=W\left(t_{20{}^{\circ}C}\right)=W_{0}\int_{x=0}^{t_{20{}^{\circ}C}}{GGR}_{20{}^{\circ}C}(x)dx$$

### Data from the literature

Some data were collected from the literature (Alkhatib and Paulsen 1984; Tashiro and Wardlaw 1990; Wardlaw *et al.* 2002; Wardlaw *et al.* 1989a,b; Zahedi *et al.* 2003; Zhao *et al.* 2007) and are summarized online **[see Supporting Information—Table S1]**. The positions of the data points were recorded in figures by image analysis (software ImageJ; http://rsbweb.nih.gov/ij/). The grain weight reductions between thermal treatments found in these studies were compared to simulations carried out with the above procedure.

## Results

### Net CO_2_ assimilation rate per unit of plant development decreased when temperature rose

**Figure 1:**
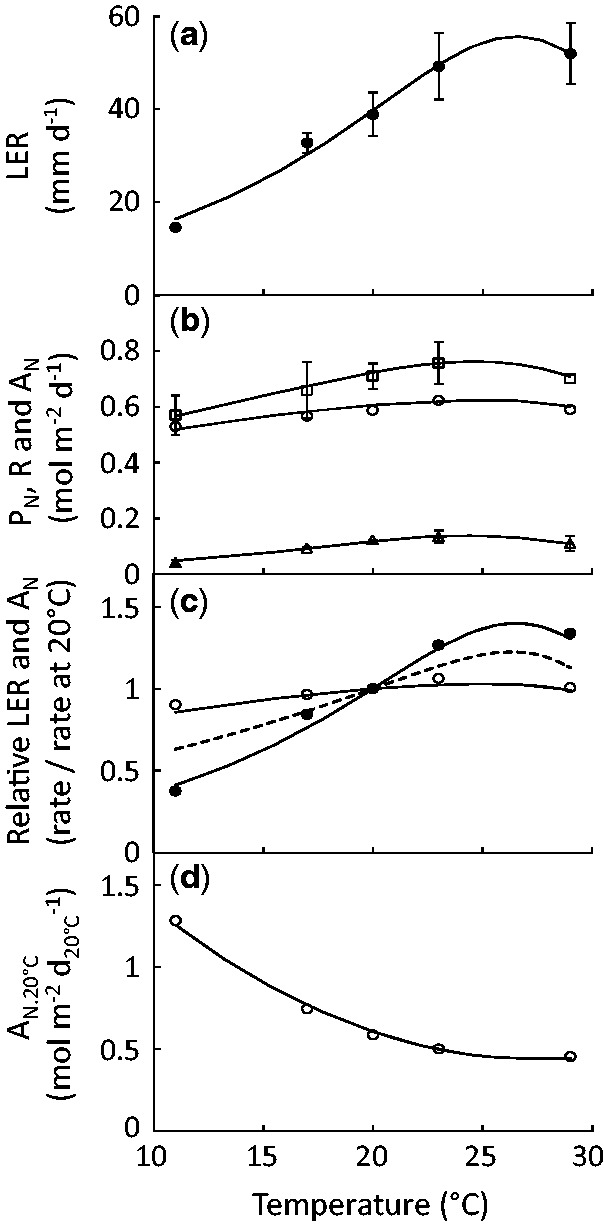
Temperature responses (experiment 1) of leaf elongation rate (LER), daily net photosynthesis (P_N_), daily dark respiration (R) and daily net CO_2_ assimilation per day (A_N_) expressed with time (d) or developmental time units (A_N.20°C_, d_20°C_). Dots: average values; error bars: confidence intervals (*p* =0.95); lines: regression from Eq.2. (**a**) LER (*n* > 8). (**b**) P_N_ (squares), R (triangles) and A_N_ (circles) (*n* > 4). (**c**) LER (black dots) and A_N_ (white dots) normalised by their respective values at 20 °C. Dashed line displays the temperature response of A_N_ under saturating light. (**d**) A_N.20°C_.

In plants where leaf 6 was emerging, rate of leaf 6 elongation (LER) was measured at five constant temperatures in the range 11 to 29 °C (Fig.1a; Experiment 1, *n* > 8). The equation of Johnson *et al.* (1942) modified by Parent and Tardieu (2012) fitted well with experimental data (Fig.1a, *R*^2 ^ = ^ ^0.99) with response parameters (Δ$H_{A}^{\text{‡}}$=69.1 kJ mol^−^^1^; *T*_0 _ = _ _29.2 °C) close to those previously determined in the meta-analysis of Parent and Tardieu (2012). The temperature response curves of net day photosynthesis (P_N_) and dark respiration (R) were also both adequately described by this equation (Fig.1b, *n* > 4, *R*^2 ^ = ^ ^0.99 and 0.97, respectively). Response of respiration was not far from that of development (Δ$H_{A}^{\text{‡}}$=74.9 kJ mol^−^^1^) but the slope of P_N_ was flatter under rising temperatures, as indicated by the low value of Δ$H_{A}^{\text{‡}}$ (19.3 kJ mol^−^^1^). When measured under saturating light, the response of photosynthesis was steeper (Δ$H_{A}^{\text{‡}}$=36.2 kJ mol^−^^1^, not shown) but still less than that of respiration or development. The temperature response curve of the net CO_2_ assimilation per day (A_N_, Fig.1b) was then calculated from P_N_ and R (Eq.1).

Temperature response curves were normalized so that they intersected the same value at 20 °C (Fig.1c), facilitating the comparison in the absence of any differences in units or magnitude (Parent *et al.* 2010a). Because leaf elongation is part of the multitude of development processes sharing a common response to temperature (Parent *et al.* 2010b; Parent and Tardieu 2012), this temperature response of normalized LER was considered as the response of development processes to temperature. It was used to adjust times and rates of other processes by the effect of temperature on general development (developmental time calculation).

The development rate accelerated more than the carbon assimilation rate as temperature increased, until the optimum temperature was reached (26.6 and 25.5 °C for LER and A_N_, respectively). Under saturating light, the two responses were more similar, although development still accelerated more than A_N_ (data not shown). Expressing A_N_ per unit of developmental time (A_N.20°C_) can be thought as an amount of carbon assimilated per standard unit of leaf elongation (and by inference, per unit of any developmental process). A_N.20°C_ decreased when the temperature rose across the measured range (Fig.1d), indicating that the amount of assimilated carbon available per unit of development decreased under rising temperatures.

### Net CO_2_ assimilation rate per unit of leaf development was linked to the dry mass per leaf area for plants grown under different thermal regimes without an additional effect of night temperature

**Figure 2:**
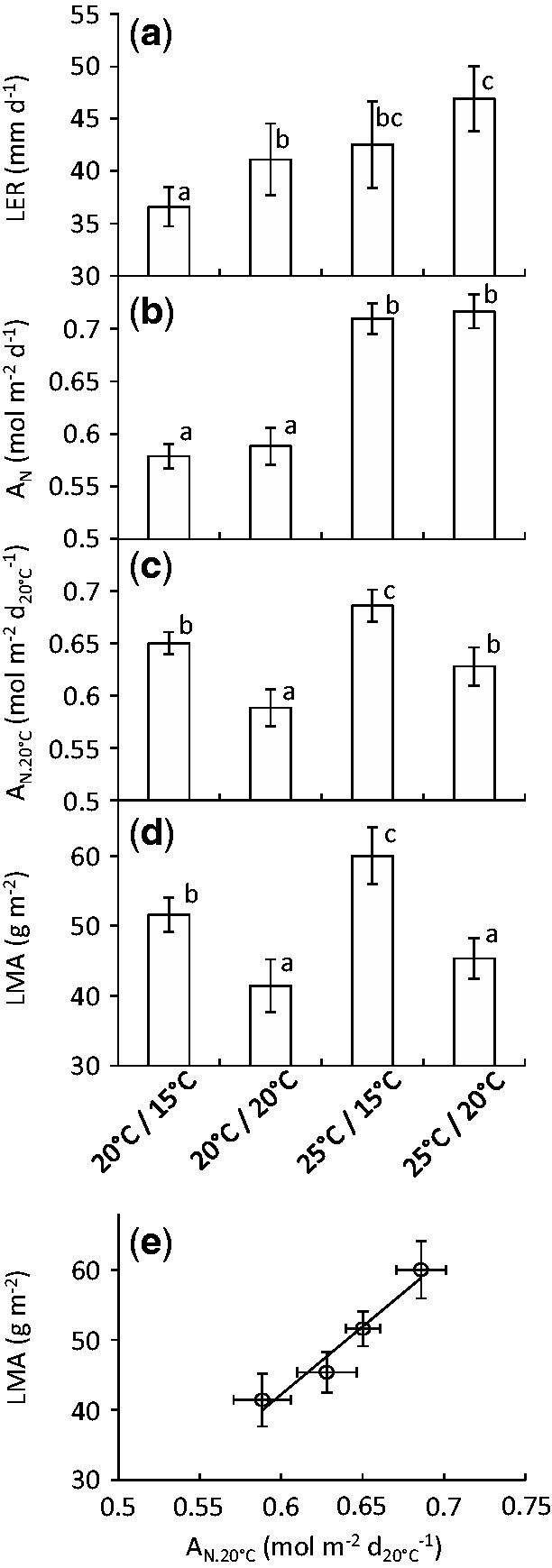
Leaf elongation rate (LER, (**a**), net CO_2_ assimilation per day (A_N_, (**b**) or day at 20 °C (A_N.20°C_, (**c**), leaf dry mass per area (LMA, averaged for leaves 4, 5, 6 and 7, (**d**) and the relationship between A_N.20°C_ and LMA (**e**) under four different temperature scenarios (T°_day_/T°_night_, experiment 2). Bars: average values (*n* = 6); error bars: confidence intervals (*p* =0.95). Means with the same letter indicate that there were no significant differences in a pairwise t-test.

Various scenarios of day/night temperature were applied at the appearance of leaf 6 to allow the net CO_2_ assimilation rate to be viewed independently of development (Fig. 2; Experiment 2, *n* = 6). LER increased about equally under increasing T°_night_ or T°_day_ (Fig. 2a), and was therefore essentially the same under thermal scenarios (T°_day_/T°_night_) 20/20 °C and 25/15 °C. By contrast, R only increased under rising T°_night_ and P_N_ only increased under rising T°_day_ [**see Supporting Information**—**Table S2**]. Because P_N_ values were much higher than R values and explained most of the variance in A_N_ (not shown), significant differences in A_N_ were only observed when T°_day_ differed (Fig. 2b). Therefore, treatment comparisons where only the night temperature differed (20/15 vs. 20/20 °C, or 25/15 vs. 25/20 °C) showed differences in LER with essentially no change in A_N_. Conversely, the comparison 25/15 vs. 20/20 °C showed differences in A_N_ with essentially no change in LER. Overall, these thermal treatments resulted in contrasting CO_2_ assimilation rates per unit of developmental time (Fig. 2c), viewed here as the amount of assimilated carbon available per unit of leaf development.

The leaf dry mass per area (LMA), measured at anthesis on leaves 4, 5, 6 and 7, was affected by thermal treatments in all leaves **[see Supporting Information—Fig. S1]** even in leaves 4 and 5, which were already partly elongated before applying the different thermal scenarios. Consequently, the average LMA in the 4 measured leaves differed significantly between treatments (Fig. 2d). These differences were mostly due to differences in leaf biomass rather than leaf area (respectively explaining 86.2 % and 2.7 % of the total variance, not shown). A temperature-induced rise in A_N_ while maintaining similar leaf expansion rate would increase the amount of assimilated carbon per unit of leaf area expansion. Accordingly, LMA was significantly greater in the 25/15 °C treatment than in the 20/20 °C treatment (60.0 ± 4.1 versus 41.4 ± 3.8 g m^−^^2^, Fig. 2d). Conversely, a temperature-induced increase in LER without any changes in A_N_ would decrease the amount of assimilated carbon per unit of leaf expansion. Accordingly, LMA was less under 20/20 °C than 20/15 °C (41.4 ± 3.8 versus 51.6 ± 2.5 g m^−^^2^), and less under 25/20 °C than 25/15 °C (45.4 ± 2.9 versus 60.0 ± 4.1g m^−^^2^). Overall, A_N.20°C_ showed a strong positive correlation with LMA (Fig. 2e, *R*^2 ^ = ^ ^0.96; *p* = 0.022 in a Pearson correlation test). Therefore, A_N.20°C_ integrated the temperature effects on leaf expansion rate and CO_2_ assimilation rate to explain differences in LMA observed between these different thermal scenarios.

### Rates of progress towards grain maturity and leaf senescence depended only on the temperature response of development

**Figure 3:**
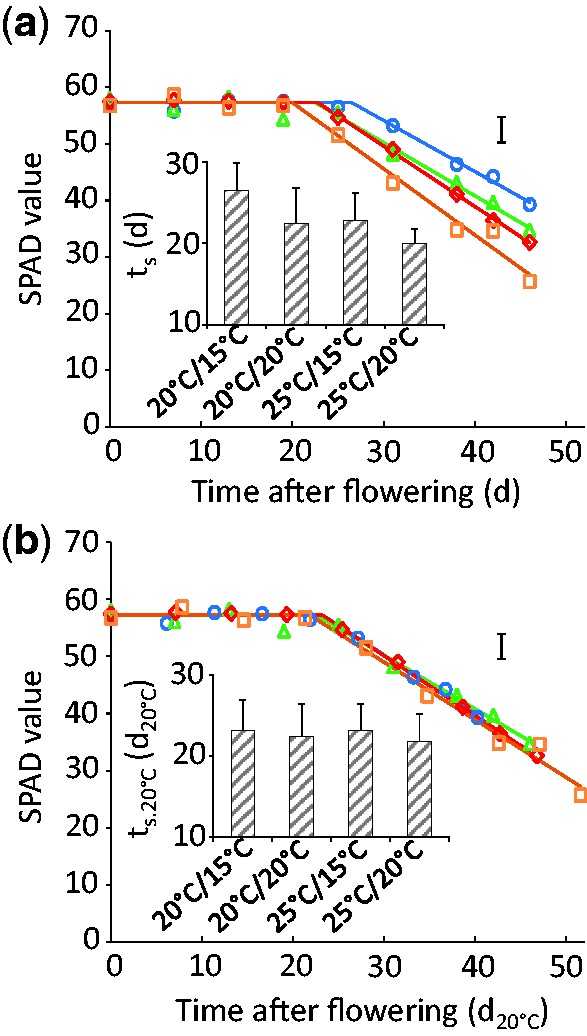
Time courses of leaf chlorophyll amount (SPAD units) under different temperature regimes (experiment 3), 20/15 °C (blue), 20/20 °C (green), 25/15 °C (red) and 25/20 °C (orange). Time is expressed either as day (d, **a**) or developmental time (d_20°C_, **b**). Dots: average values (*n*≥ 4). Error bar: average confidence intervals (*p* = 0.95). Lines are bilinear regressions with 3 parameters (SPAD_0_, t_s_, a_s_). SPAD_0_ is fixed and common to all treatments. **Inset in a)** Values of t_s_. Bars: parameter value ± confidence interval calculated by bootstrap (*p* = 0.95). **Inset in b)** Values of t_s.20°C_. Bars: parameter value ± confidence interval (*p* = 0.95).

**Figure 4:**
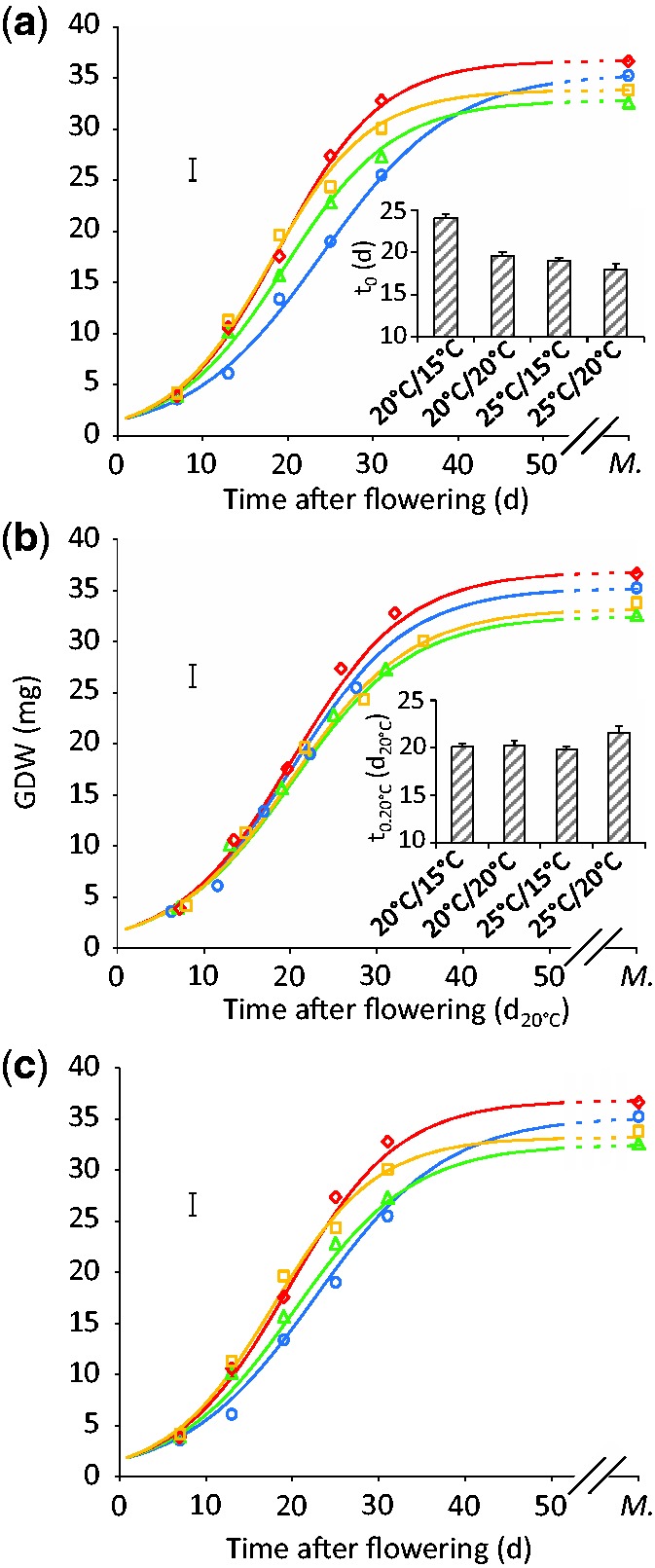
Time courses of individual grain dry weight (GDW) under different temperature regimes (experiment 3), 20/15 °C (blue), 20/20 °C (green), 25/15 °C (red), 25/20 °C (orange). Time is expressed either as days (**a**, **c**) or developmental time (d_20°C_, **b**). *M*.: grain maturity. Dots: average values (*n*≥ 4). Error bars: average confidence intervals (*P* = 0.95). Lines are logistic regressions with 3 parameters (W_0_, *t*_0_, λ). W_0_ is fixed and common to all treatments. (**a)** λ and *t*_0_ are free in each treatment. **Inset in a)** values of *t*_0 _± confidence interval (*p* = 0.95). (**b**) λ and *t*_0_ are free in each treatment but with time expressed as developmental time (d_20°C_). **Inset in b)** values of *t*_0_20°C _±_ _confidence interval (*P* = 0.95). (**c**) λ is the only free parameter in each treatment. *t*_0_ (**d**) is calculated in each treatment from a single *t*_0_20°C_ value (d_20°C_), common to all treatments.

Plants at anthesis were introduced to several temperature scenarios, and then leaf senescence and biomass accumulation in the grain were measured over time (Fig. 3a and Fig. 4a; Experiment 3; *n* > 4 for each time point). Chlorophyll content in the three last developed leaves, defined in SPAD units, was at first stable, and then decreased linearly. Fitting a bilinear model enabled the calculation of the time at which the chlorophyll level started to decrease (t_s_). This parameter was closely correlated with the average daily temperatures (from 20.0 ± 1.7 at 25/20 °C to 26.5 ± 3.4 d at 20/15 °C, Fig. 3a inset). When time and model parameters were expressed in developmental time units (Fig. 3b), profiles of leaf senescence were similar between thermal treatments (*t*_s.20°C_ ranging from 21.8 ± 3.4 to 23.2 ± 3.7 d_20°C_; Fig. 3b inset).

Fitting logistic curves (Eq.5) to the time courses of single grain dry weight (GDW; Fig. 4a) resulted in various values of *t*_0_, the time at which grain weight reached half of the final dry weight and growth was maximal (Fig. 4a inset). Its values decreased with rising average temperatures (from 24.0 ± 0.5 to 17.9 ± 0.7 d). Similarly, the time taken for complete grain fill (t_f_) decreased by 11 d with rising temperatures (from 46.6 to 35.1 d, not shown). However, grain filling duration was similar in the 25/15 and 20/20 °C treatments (36.8 d and 38.4 d, not shown) indicating that it was largely independent of carbon assimilation. When time was expressed in developmental time units (d_20°C_, Fig. 4b), values of *t*_0_20°C_ were similar across treatments (ranging from 19.8 ± 0.3 to 21.6 ± 0.7 d_20°C_, Fig. 4b inset) as were the values of grain filling duration (from 39.2 to 42.3 d_20°C_, not shown).

Overall, rates toward grain maturity and rates of leaf senescence were similar across thermal treatments when expressed in developmental time. Grain filling duration was only dependent on average temperature, and mostly independent of carbon supply.

### Maximum rates of biomass accumulation in individual grains were dependent on net CO_2_ assimilation but independent of development rates

The time courses of biomass accumulation in the grain were adequately described by the logistic model when only one parameter (λ) was kept free in each thermal scenario (W_0_ and *t*_0_20°C_ fixed in all treatments, Fig. 4c, *t*_0_20°C _ =  20.2 d_20°C_; see Material and Methods **[see Supporting Information—Methods S1]**).

**Figure 5:**
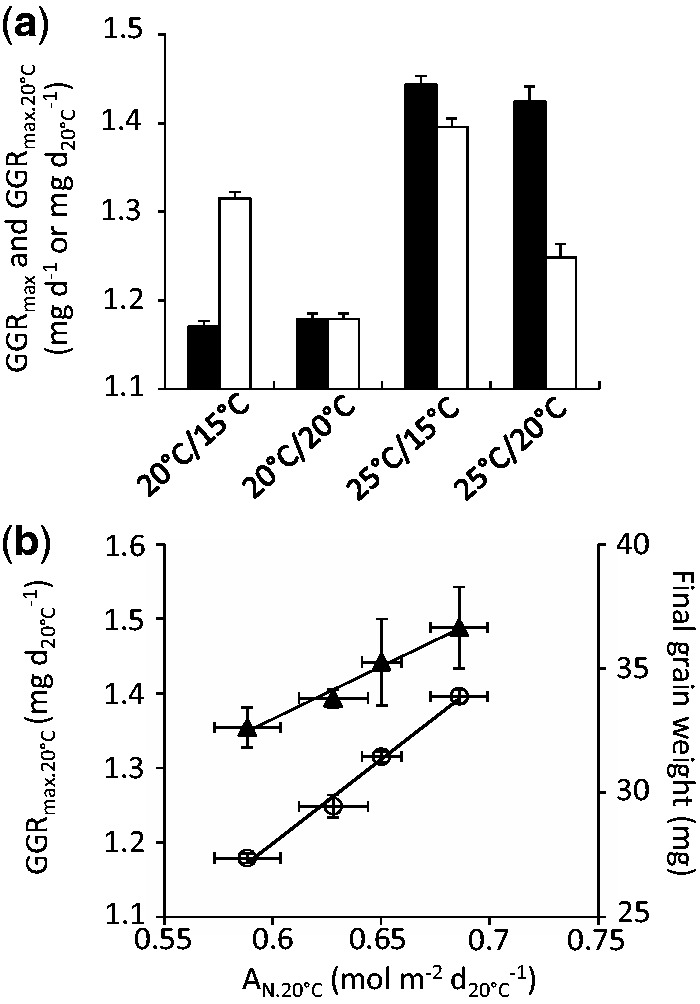
Values of maximum grain growth rate (GGR_max_, (**a**) estimated from regression displayed in Fig.4c (W_0_ and *t*_0_ fixed), expressed with time (black bars) or developmental time units (white bars), and the relationship between net CO_2_ assimilation per d_20°C_ (A_N.20°C_) and final individual grain weight or GGR_max.20°C_ in the 4 different temperature scenarios (**b**). (**a**) Bars: estimated parameter value. Error bar: confidence interval (*P* = 0.95). (**b**) Grey triangles: final grain weight. White circles: GGR_max.20°C_. A_N.20°C_ values were measured in Experiment 2 and shown in Fig. 2 **[see Supporting Information**—**Table S2]**.

As the maximum rate of accumulation of dry weight in single grains (GGR_max_) and λ are interdependent variables (Eq.6), grain growth responses to temperature are hereafter described in terms of GGR_max_ only (more intuitive than λ). GGR_max_ varied between thermal treatments, especially where day temperature differed (Figs 4c and 5a). Because temperature accelerated leaf senescence and progress towards grain maturity similarly, effects of temperature on rates of grain dry weight accumulation could not be attributed to one or the other of these factors.

Relative to the 25/15 °C treatment, the 20/20 °C treatment showed an increase in CO_2_ assimilation (A_N_) and GGR_max_ (1.18 ± 0.01 to 1.44 ± 0.02 mg d^−^^1^, Fig. 5a) but a similar rate of progress toward grain maturity. By contrast, increasing night temperature, *i.e.* 20/15 vs. 20/20 °C, or 25/15 vs. 25/20 °C, increased development rate but not A_N_ or GGR_max_ (Fig. 5a). Therefore, GGR_max_ appeared to be only dependent on carbon assimilation rate and largely independent of development rate.

Overall, the two contributors to final grain weight, the rate toward grain maturity and the rate of biomass accumulation in the grain, behaved independently, and correlated with temperature responses of development and of carbon assimilation, respectively.

### Net CO_2_ assimilation rate expressed in developmental units explained the differences in dynamics of grain biomass accumulation

When expressed in developmental units, maximum grain growth rate (GGR_max.20°C_, Fig. 5a) was dependent on both the rate of development and of CO_2_ assimilation. GGR_max.20°C_ can be thought as the biomass accumulation per standard unit of grain development. In the same way, A_N_ expressed per unit of developmental time (A_N.20°C_) can be thought as the amount of assimilated carbon available per unit of grain development. An increase in CO_2_ assimilation for a similar grain development rate increased GGR_max.20°C_ (20/20 *vs*. 25/15 °C; 1.18 to 1.40 mg d_20°C_^−^^1^, Fig. 5a). Increasing the grain development rate without increasing the CO_2_ assimilation rate resulted in lower GGR_max.20°C_, as shown in treatments 20/15 *vs*. 20/20 °C or 25/15 *vs*. 25/20 °C, Fig. 5a). A_N.20°C_ was positively correlated with GGR_max.20°C_ (Fig. 5b, *R*^2 ^ = ^ ^0.97, *p* = 0.009 in a Pearson correlation test). Because GGR_max.20°C_ could completely describe the time course of biomass accumulation, A_N.20°C_ was correlated with final grain weight (Fig. 5b, *R*^2 ^ = ^ ^0.98, *p* = 0.005 in a Pearson correlation test).

Overall, by integrating the temperature effects on the rates of grain development and CO_2_ assimilation, A_N.20°C_ was able to explain the differences in the grain growth rate and final grain weight observed between the different thermal scenarios.

**Figure 6:**
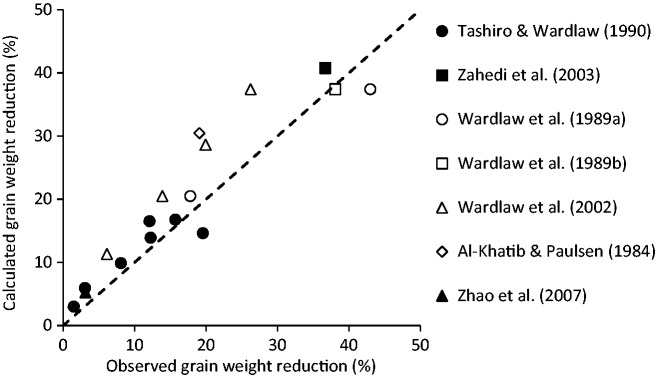
Observed values *vs.* calculated values for the reduction in final grain weight between temperatures treatments. Observed data come from the literature **[see Supporting Information—Table S1]**. Dashed line is the model x = y.

This relationship was used to simulate final grain weight effects reported in seven different papers for various thermal scenarios involving T°_day_ up to 30 °C and T°_night_ up to 25 °C (Fig. 6). The predicted grain weight reductions were not far from the observed ones (*R*^2 ^ = ^ ^0.79), suggesting that the relationship between A_N.20°C_ and grain growth rate could hold true for other genotypes, environmental conditions, and thermal scenarios within the investigated range. However, the model had a tendency to over-estimate the negative effect of rising temperatures (average bias of 16%), indicating a genetic variability for this relationship, or the influence of other physiological processes such as carbon remobilization to the grains.

## Discussion

### Temperature response patterns of biomass accumulation in leaves and grains as a consequence of the discrepancy between development and carbon assimilation responses

Various studies have emphasized a role of altered carbon supply-demand in the effects of high temperature on plant processes (Taub *et al.* 2000; Vasseur *et al.* 2011; Vile *et al.* 2012). Yet, this concept has rarely been tested by concurrently monitoring temperature responses of development, carbon assimilation and biomass accumulation (Poorter *et al.* 2009), or in a range of temperatures that were not harmful to photosynthesis (Vasseur *et al.* 2011; Vile *et al.* 2012). Therefore, we simultaneously monitored the temperature responses of development, respiration and photosynthesis in the non-stressing range. These responses were divergent, resulting in a variation in carbon supply relative to development across various thermal treatments. Under rising temperatures, an increase in photosynthesis increased both LMA and grain weight, while accelerated development reduced leaf and grain weights. We showed that the discrepancy between the temperature responses of development and carbon assimilation could explain the observed patterns of biomass accumulation in wheat leaves and grains across a range of thermal scenarios.

### Expressing net CO_2_ assimilation and biomass accumulation per unit of development summarizes the effects of temperature on development and carbon assimilation

Here, we examined the possibility of using the thermal compensation of time and rates to dissect the factors influencing biomass accumulation. Previously, this concept was applied to enable the effects of other environmental variables on leaf expansion (Parent *et al.* 2010b), cell expansion profiles in leaf (Parent *et al.* 2009) or endogenous rhythms (Poire *et al.* 2010) to be studied independently of the effect of temperature on development. In the current study, by expressing the rates of processes not classified as ‘development processes’, such as biomass accumulation in tissues, in terms of rate per unit of development, we were able to quantify the component of the biomass accumulation response that was controlled purely by fluctuations in net carbon assimilation. Expressing the net assimilation rate in terms of developmental time therefore summarized the effects of temperature on photosynthesis, respiration and development. It can be thought as the ratio of the source/development sink, or as the amount of assimilated carbon available per unit of plant development. In addition, a simple model using this trait as the indicator of source-sink dynamics was able to explain most of the effects of thermal scenarios on grain weight, across different genotypes and environmental conditions.

By allowing the contribution of net carbon fixation on biomass accumulation across a temperature range to be followed independently of the effect of temperature on development, this approach makes possible an assessment of the impact of other factors (*e.g.* light intensity) on biomass accumulation across a range of temperatures. Furthermore, it could provide an approach for quantifying longer lasting heat damage caused by factors such as protein denaturation that are likely encountered at much higher temperatures, independent of reversible responses of a purely thermodynamic nature.

### Rising night temperature is likely to decrease biomass production

Increasing either night or day temperature would accelerate development by the same degree (Morita *et al.* 2005; Parent *et al.* 2010a), but only increases in T°_night_ would increase respiration without any compensatory increase in photosynthesis. Simple simulations also indicate that A_N.20°C_ would be more sensitive to an increase in T°_night_ than to a similar increase in T°_day_ or the 24-h average temperature T°_ave_ (not shown). Indeed, our own experiment employing four day/night thermal treatments demonstrated that increasing T°_night_ reduced grain biomass more than increasing T°_day_ or T°_ave_. In the simulation shown in **Supporting Information—Fig. S2**, increasing night temperature by 5 °C decreased A_N.20°C_ from 1.33 to 1.09 mol m^−^^2^ d_20°C_^−1^ (not shown) and therefore decreased final grain weight by 15.3 %.

The effect of maximum daily temperature (T_max_) and minimum daily temperature (T_min_; which occurs during the night) on the performance of wheat and rice in the field has been examined using data across multiple environments. Such studies have revealed greater and more frequent negative impacts of warming during the night than warming during the day (Peng *et al.* 2004; Welch *et al.* 2010; Lobell and Ortiz-Monasterio 2007; Cossani and Reynolds 2012). Our findings offer a potential explanation for these differential effects of day and night temperature on crop productivity in the field. In this study, no additional ‘hidden’ effect of night temperature was detected.

### *Could temperature acclimation change this pattern*?

While temperature changes in the non-stressing range can perturb photosynthesis and respiration in the short-term, the rates of these two processes can eventually recover completely, due to acclimation (Atkin *et al.* 2006; Campbell *et al.* 2007). Acclimation might make net CO_2_ assimilation insensitive to any long-term temperature change (Atkin *et al.* 2006). By contrast, development rate was found to be stably dependent on temperature, and did not acclimate (Parent and Tardieu 2012). Therefore, it is possible that long term responses of biomass accumulation to rising temperature, such as those experienced across the seasons, may only depend on the temperature responses of development, resulting in a greater reduction in biomass (mass per unit of development) than is predicted from the presented model. The model may apply better to day to day fluctuations, such as brief heat waves of several days duration, which commonly occur in the southern Australian wheat belt during the flowering and grain filling period and correlate with significant grain yield losses (Wardlaw and Wrigley 1994).

### Diversity of biomass accumulation responses

The temperature response of CO_2_ assimilation per unit of plant development can present a large diversity. Firstly, there is a large diversity between plant species for the temperature responses of photosynthesis and respiration rates (Loveys *et al.* 2002), as well as for temperature acclimation of these processes (Atkin *et al.* 2006). In addition, there is a large genetic variability for development rate *per se* (Borras-Gelonch *et al.* 2010). The temperature response of development, while highly conserved in each species presented also a large variability between species (Parent and Tardieu 2012). It follows that the overall response of the net assimilation per unit of plant development could present a large diversity between genotypes or species.

Grain biomass and yield in a broad sense do not depend only on the total assimilated carbon. A large genetic variability can be found in the ability of plants to mobilize and allocate carbon to the grains (Reynolds *et al.* 2009). It probably explains why the model over-estimated the effects of temperature on grain size in Fig. 6. These processes have their own response to temperature (Poorter *et al.* 2012) and can therefore present interesting genetic variability. In wheat, improving photosynthesis efficiency and partitioning to the grain are the central targets of the International Wheat Consortium (IWC, Reynolds *et al.* 2011).

The presented model was intentionally simple, used only to test the presented hypothesis, that the discrepancy between CO_2_ assimilation and development responses were responsible for the response of biomass accumulation in tissues. However, the diversity of underlying physiological processes presented above would result in a wide diversity of carbon assimilation per unit of plant development. Experimenters need to be aware of these factors, and this model should be built on or adjusted to account for them, to suit any particular experimental system.

## Conclusion

Models based on data collected under controlled conditions were developed to predict net CO_2_ assimilation rate per unit of plant development under various temperature scenarios. This unit for expressing biomass accumulation rate (i) summarized the effect of the temperature responses of development, respiration and photosynthesis, (ii) provided a means of comparing rates of biomass accumulation obtained under different growth conditions, independent of the effects of temperature on development, and (iii) represents a potential approach for quantifying irreversible versus reversible responses that may occur in the extremely high temperature range. The model is likely to require modification under certain circumstances, *e.g.* where acclimation, photosynthate mobilization processes, and genotypic variation are additional factors in temperature responses.

## Sources of Funding 

This work was supported by the European projects FP7-244374 (DROPS) and FP7-613817 (MODEXTREME) and the Grains Research and Development Corporation (GRDC) project UA00123. ACPFG was also funded by the GRDC, the Australian Research Council, the Government of South Australia and the University of Adelaide.

## Contributions by the Authors

Iman Lohraseb carried out most experiments; Nicholas C. Collins contributed to interpretation of the data and preparation of the manuscript; Boris Parent performed most analyses and prepared the manuscript

## Conflict of Interest Statement

None declared.

## Supplementary Material

Supplementary Data

## References

[plw092-B1] Alkhatib K Paulsen GM. 1984 Mode of high-temperature injury to wheat during grain development. Physiologia Plantarum 61:363–368.

[plw092-B2] Atkin OK Scheurwater I Pons TL. 2006 High thermal acclimation potential of both photosynthesis and respiration in two lowland *Plantago* species in contrast to an alpine congeneric. Global Change Biology 12:500–515.

[plw092-B3] Atkin OK Scheurwater I Pons TL. 2007 Respiration as a percentage of daily photosynthesis in whole plants is homeostatic at moderate, but not high, growth temperatures. New Phytologist 174:367–380. 17388899 10.1111/j.1469-8137.2007.02011.x

[plw092-B4] Atkin OK Tjoelker MG. 2003 Thermal acclimation and the dynamic response of plant respiration to temperature. Trends in Plant Science 8:343–351. 12878019 10.1016/S1360-1385(03)00136-5

[plw092-B9] Borras-Gelonch G Slafer GA Casas AM van Eeuwijk F Romagosa I. 2010 Genetic control of pre-heading phases and other traits related to development in a double-haploid barley (*Hordeum vulgare* L.) population. Field Crops Research 119:36–47.

[plw092-B10] Campbell C Atkinson L Zaragoza-Castells J Lundmark M Atkin O Hurry V. 2007 Acclimation of photosynthesis and respiration is asynchronous in response to changes in temperature regardless of plant functional group. New Phytologist 176:375–389. 17692077 10.1111/j.1469-8137.2007.02183.x

[plw092-B11] O'Connell MG O'Leary GJ Whitfield DM Connor DJ. 2004 Interception of photosynthetically active radiation and radiation-use efficiency of wheat, field pea and mustard in a semi-arid environment. Field Crops Research 85:111–124.

[plw092-B12] Cossani CM Reynolds MP 2012 Physiological Traits for Improving Heat Tolerance in Wheat. Plant Physiology 160:1710–1718. 23054564 10.1104/pp.112.207753 PMC3510104

[plw092-B13] Fang S Cammarano D Zhou G Tan K Ren S. 2015 Effects of increased day and night temperature with supplemental infrared heating on winter wheat growth in North China. European Journal of Agronomy 64:67–77.

[plw092-B15] Glaubitz U Li X Koehl KI van Dongen JT Hincha DK Zuther E. 2014 Differential physiological responses of different rice (*Oryza sativa*) cultivars to elevated night temperature during vegetative growth. Functional Plant Biology 41:437–448. 10.1071/FP13132 32481003

[plw092-B16] Johnson FH Eyring H Williams RW. 1942 The nature of enzyme inhibitions in bacterial luminescence: Sulfanilamide, urethane, temperature and pressure. Journal of Cellular and Comparative Physiology 20:247–268.

[plw092-B17] Kanno K Makino A. 2010 Increased grain yield and biomass allocation in rice under cool night temperature. Soil Science and Plant Nutrition 56:412–417.

[plw092-B19] Kumudini S Andrade FH Boote KJ Brown GA Dzotsi KA Edmeades GO Gocken T Goodwin M Halter AL Hammer GL Hatfield JL Jones JW Kemanian AR Kim SH Kiniry J Lizaso JI Nendel C Nielsen RL Parent B Stoeckle CO Tardieu F Thomison PR Timlin DJ Vyn TJ Wallach D Yang HS Tollenaar M. 2014 Predicting Maize Phenology: Intercomparison of Functions for Developmental Response to Temperature. Agronomy Journal 106:2087–2097.

[plw092-B60] Lobell DB Ortiz-Monasterio JI. 2007 Impacts of day versus night temperatures on spring wheat yields: A comparison of empirical and CERES model predictions in three locations. Agronomy Journal 99:469–477.

[plw092-B20] Louarn G Andrieu B Giauffret C. 2010 A size-mediated effect can compensate for transient chilling stress affecting maize (*Zea mays*) leaf extension. New Phytologist 187:106–118. 20456066 10.1111/j.1469-8137.2010.03260.x

[plw092-B21] Loveys BR Scheurwater I Pons TL Fitter AH Atkin OK. 2002 Growth temperature influences the underlying components of relative growth rate: an investigation using inherently fast- and slow-growing plant species. Plant Cell and Environment 25:975–987.

[plw092-B22] Makowski D Asseng S Ewert F Bassu S Durand JL Li T Martre P Adam M Aggarwal PK Angulo C Baron C Basso B Bertuzzi P Biernath C Boogaard H Boote KJ Bouman B Bregaglio S Brisson N Buis S Cammarano D Challinor AJ Confalonieri R Conijn JG Corbeels M Deryng D De Sanctis G Doltra J Fumoto T Gaydon D Gayler S Goldberg R Grant RF Grassini P Hatfield JL Hasegawa T Heng L Hoek S Hooker J Hunt LA Ingwersen J Izaurralde RC Jongschaap REE Jones JW Kemanian RA Kersebaum KC Kim SH Lizaso J Marcaida M III Mueller C Nakagawa H Kumar SN Nendel C O'Leary GJ Olesen JE Oriol P Osborne TM Palosuo T Pravia MV Priesack E Ripoche D Rosenzweig C Ruane AC Ruget F Sau F Semenov MA Shcherbak I Singh B Singh U Soo HK Steduto P Stoeckle C Stratonovitch P Streck T Supit I Tang L Tao F Teixeira EI Thorburn P Timlin D Travasso M Roetter RP Waha K Wallach D White JW Wilkens P Williams JR Wolf J Yin X Yoshida H Zhang Z Zhu Y. 2015 A statistical analysis of three ensembles of crop model responses to temperature and CO_2_ concentration. Agricultural and Forest Meteorology 214:483–493.

[plw092-B23] Morita S Yonemaru J Takanashi J. 2005 Grain growth and endosperm cell size under high night temperatures in rice (*Oryza sativa* L.). Annals of Botany 95:695–701. 15655104 10.1093/aob/mci071 PMC4246861

[plw092-B24] Parent B Conejero G Tardieu F. 2009 Spatial and temporal analysis of non-steady elongation of rice leaves. Plant Cell and Environment 32:1561–1572. 10.1111/j.1365-3040.2009.02020.x 19627567

[plw092-B25] Parent B Suard B Serraj R Tardieu F. 2010b Rice leaf growth and water potential are resilient to evaporative demand and soil water deficit once the effects of root system are neutralized. Plant Cell and Environment 33:1256–1267. 10.1111/j.1365-3040.2010.02145.x 20302604

[plw092-B26] Parent B Tardieu F. 2012 Temperature responses of developmental processes have not been affected by breeding in different ecological areas for 17 crop species. The New Phytologist 194:760–774. 22390357 10.1111/j.1469-8137.2012.04086.x

[plw092-B27] Parent B Tardieu F. 2014 Can current crop models be used in the phenotyping era for predicting the genetic variability of yield of plants subjected to drought or high temperature?. Journal of Experimental Botany 65:6179–6189. 24948682 10.1093/jxb/eru223

[plw092-B28] Parent B Turc O Gibon Y Stitt M Tardieu F. 2010a Modelling temperature-compensated physiological rates, based on the co-ordination of responses to temperature of developmental processes. Journal of Experimental Botany 61:2057–2069. 20194927 10.1093/jxb/erq003

[plw092-B29] Peng SB Huang JL Sheehy JE Laza RC Visperas RM Zhong XH Centeno GS Khush GS Cassman KG. 2004 Rice yields decline with higher night temperature from global warming. Proceedings of the National Academy of Sciences of the United States of America 101:9971–9975. 15226500 10.1073/pnas.0403720101 PMC454199

[plw092-B30] Peraudeau S Rogues S Quinones CO Fabre D Van Rie J Ouwerkerk PBF Jagadish KSV Dingkuhn M Lafarge T. 2015 Increase in night temperature in rice enhances respiration rate without significant impact on biomass accumulation. Field Crops Research 171:67–78.

[plw092-B31] Poire R Wiese-Klinkenberg A Parent B Mielewczik M Schurr U Tardieu F Walter A. 2010 Diel time-courses of leaf growth in monocot and dicot species: endogenous rhythms and temperature effects. Journal of Experimental Botany 61:1751–1759. 20299442 10.1093/jxb/erq049 PMC2852670

[plw092-B32] Poorter H Niinemets U Poorter L Wright IJ Villar R. 2009 Causes and consequences of variation in leaf mass per area (LMA): a meta-analysis. New Phytologist 182:565–588. 19434804 10.1111/j.1469-8137.2009.02830.x

[plw092-B33] Poorter H Niklas KJ Reich PB Oleksyn J Poot P Mommer L. 2012 Biomass allocation to leaves, stems and roots: meta-analyses of interspecific variation and environmental control. New Phytologist 193:30–50. 22085245 10.1111/j.1469-8137.2011.03952.x

[plw092-B34] R Development Core Team. 2005. R: A language and environment for statistical computing, reference index version 2.2.1. R Foundation for statistical Computing, Vienna, Austria

[plw092-B35] Reynolds M Bonnett D Chapman SC Furbank RT Manes Y Mather DE Parry MAJ. 2011 Raising yield potential of wheat. I. Overview of a consortium approach and breeding strategies. Journal of Experimental Botany 62:439–452. 20952629 10.1093/jxb/erq311

[plw092-B36] Reynolds M Foulkes MJ Slafer GA Berry P Parry MAJ Snape JW Angus WJ. 2009 Raising yield potential in wheat. Journal of Experimental Botany 60:1899–1918. 19363203 10.1093/jxb/erp016

[plw092-B37] Rosenzweig C Jones JW Hatfield JL Ruane AC Boote KJ Thorburn P Antle JM Nelson GC Porter C Janssen S Asseng S Basso B Ewert F Wallach D Baigorria G Winter JM. 2013 The Agricultural Model Intercomparison and Improvement Project (AgMIP): Protocols and pilot studies. Agricultural and Forest Meteorology 170:166–182.

[plw092-B39] Sage RF Kubien DS. 2007 The temperature response of C-3 and C-4 photosynthesis. Plant Cell and Environment 30:1086–1106. 10.1111/j.1365-3040.2007.01682.x 17661749

[plw092-B40] Sofield I Evans LT Cook MG Wardlaw IF. 1977 Factors influencing rate and duration of grain filling in wheat. Australian Journal of Plant Physiology 4:785–797.

[plw092-B41] Tardieu F Granier C Muller B. 1999 Modelling leaf expansion in a fluctuating environment: are changes in specific leaf area a consequence of changes in expansion rate?. New Phytologist 143:33–44.

[plw092-B42] Tashiro T Wardlaw IF. 1990 The effects of high temperature at different stages of ripening on garin set, grain weight and grain dimensions in the semi dwarf wheat “Banks”. Annals of Botany 65:51–61.

[plw092-B43] Taub DR Seemann JR Coleman JS. 2000 Growth in elevated CO_2_ protects photosynthesis against high-temperature damage. Plant Cell and Environment 23:649–656.

[plw092-B44] Vasseur F Pantin F Vile D. 2011 Changes in light intensity reveal a major role for carbon balance in Arabidopsis responses to high temperature. Plant Cell and Environment 34:1563–1576. 10.1111/j.1365-3040.2011.02353.x 21707647

[plw092-B45] Vile D Pervent M Belluau M Vasseur F Bresson J Muller B Granier C Simonneau T. 2012 Arabidopsis growth under prolonged high temperature and water deficit: independent or interactive effects?. Plant Cell and Environment 35:702–718. 10.1111/j.1365-3040.2011.02445.x 21988660

[plw092-B46] Wardlaw IF. 1994 The effect of high temperature on kernel development in wheat: Variability related to pre-heading and postanthesis conditions. Australian Journal of Plant Physiology 21:731–739.

[plw092-B47] Wardlaw IF Blumenthal C Larroque O Wrigley CW. 2002 Contrasting effects of chronic heat stress and heat shock on kernel weight and flour quality in wheat. Functional Plant Biology 29:25–34. 10.1071/PP00147 32689448

[plw092-B48] Wardlaw IF Dawson IA Munibi P. 1989a The tolerance of wheat to high temperatures during reproductive growth. 2. Grain development. Australian Journal of Agricultural Research 40:15–24.

[plw092-B49] Wardlaw IF Dawson IA Munibi P Fewster R. 1989b The tolerance of wheat to high temperatures during reproductive growth. 1. Survey procedures and general response patterns. Australian Journal of Agricultural Research 40:1–13.

[plw092-B50] Wardlaw IF Wrigley CW. 1994 Heat tolerance in temperate cereals. An overview. Australian Journal of Plant Physiology 21:695–703.

[plw092-B52] Welch JR Vincent JR Auffhammer M Moya PF Dobermann A Dawe D. 2010 Rice yields in tropical/subtropical Asia exhibit large but opposing sensitivities to minimum and maximum temperatures. Proceedings of the National Academy of Sciences of the United States of America 107:14562–14567. 20696908 10.1073/pnas.1001222107 PMC2930450

[plw092-B53] Wheeler TR Hong TD Ellis RH Batts GR Morison JIL Hadley P. 1996 The duration and rate of grain growth, and harvest index, of wheat (*Triticum aestivum* L) in response to temperature and CO_2_. Journal of Experimental Botany 47:623–630.

[plw092-B54] Yin X Guo W Spiertz JH. 2009 A quantitative approach to characterize sink-source relationships during grain filling in contrasting wheat genotypes. Field Crops Research 114:119–126.

[plw092-B55] Zahedi M Sharma R Jenner CF. 2003 Effects of high temperature on grain growth and on the metabolites and enzymes in the starch-synthesis pathway in the grains of two wheat cultivars differing in their responses to temperature. Functional Plant Biology 30:291–300. 10.1071/FP02205 32689011

[plw092-B56] Zhao H Dai T Jing Q Jiang D Cao W. 2007 Leaf senescence and grain filling affected by post-anthesis high temperatures in two different wheat cultivars. Plant Growth Regulation 51:149–158.

