# Supplementary material for: Diverging temperature responses of CO2 assimilation and plant development explain the overall effect of temperature on biomass accumulation in wheat leaves and grains
Source: AoB Plants. 2017 Jan 9;9(1):plw092. doi: 10.1093/aobpla/plw092 (PMC5391697; doi:10.1093/aobpla/plw092)
Supplement: Supplementary Data [file plw092_Supp.pdf]

Supplementary Information
Method S1

Method S1. Fitting procedures and parameters obtained for leaf senescence or growth of individual grain weight.

Fitting procedure for leaf senescence.

In each thermal scenario, a bilinear model was fitted to the dataset. It consisted in a constant value ($SPAD_0$) until a time of senescence (t_s) and a linear decrease in chlorophyll content after this point (slope a_s). Because plants had a similar thermal treatment before anthesis, $SPAD_0$ was fixed for all thermal scenarios and equalled the average value in all treatments at anthesis ($SPAD_0$=57.3). A set of t_s values was tested from 16 d to 28 d. For each value of $t_{s.}$, the last free parameter a_s was determined by linear regression, and the coefficient of determination R^2 was calculated for the whole dataset (plateau and linear decrease). The best value of t_s was the one minimising the coefficient of determination R^2. A similar procedure was carried out considering time *t* and t_s as developmental time ($t_{20°C}$ and $t_{s.20°C}$, $d_{20°C}$).

Logistic equation for fitting

A well-known version of the logistic equation, already used for modelling individual grain growth (Morita et al., 2005) is

$$W(t) = \frac{\mathrm{W_f}}{1 + \mathrm{e}^{(-\lambda\,(t - \mathrm{t_0}))}}$$

with *W(t)* the individual grain weight at time *t* after anthesis; W_f the final weight, λ is the slope factor controlling the steepness of the curve and t_0 the inflexion point, or time at which seed weight reached the half of final weight. This equation was modified to get the theoretical grain weight at anthesis (W_0) as parameters of the equation. At t=0:

$$W(0) = \mathrm{W_0} = \frac{\mathrm{W_f}}{1 + \mathrm{e}^{(\lambda\,\mathrm{t_0})}}$$

$$\mathrm{W_f} = \mathrm{W_0}\left(1 + \mathrm{e}^{(\lambda\,\mathrm{t_0})}\right)$$

It followed the final version of the logistic equation used in this study for fitting grain growth.

$$W(t) = \frac{\mathrm{W_0}\left(1 + \mathrm{e}^{(\lambda\,\mathrm{t_0})}\right)}{1 + \mathrm{e}^{(-\lambda\,(t - \mathrm{t_0}))}}$$

Fitting procedure for the logistic equation

This equation was fitted in each thermal scenario by using the non-linear regression function *nls*. W_0 varied from 1.4 to 2.0 mg depending on treatment. Because plants were transferred in the different thermal treatments at anthesis, W_0 was considered as common in all treatments. A set of values for W_0 was then tested from 1.4 to 2.0 mg with a step of 0.05 mg, and the chosen value was the one minimising the coefficient of determination R^2 for the whole dataset (all treatments together) after regression in each thermal treatment (W_0=1.65 mg).
Eq.5 was fitted to each dataset, considering time either as days or developmental time ($t_{20°C}$, $d_{20°C}$). In the last case, the two free parameters are expressed with developmental time units ($t_{0.20°C}$, $d_{20°C}$; $\lambda_{20°C}$, $d_{20°C}^{-1}$).
To determine a unique $t_{0.20°C}$ value common to all treatments, a set of $t_{0.20°C}$ values was tested from 16 $d_{20°C}$ to 25 $d_{20°C}$ with a step of 0.1 $d_{20°C}$. For each value of $t_{0.20°C}$, the last free parameter $\lambda_{20°C}$ was determined by non-linear regression in each treatment. The value of $t_{0.20°C}$ considered as common to all thermal treatments was the one minimising R^2 for the whole dataset (all treatments together). Corresponding values of t_0 (d) in each treatment were calculated from Eq.4 and $t_{0.20°C}$. Similarly, in each thermal treatment, λ can be calculated from Eq.3 and $\lambda_{20°C}$.

Calculation of grain growth rate

The grain growth rate (*GGR)* at each time t, was obtained by derivation of Eq.5, with time and model parameters expressed either with time or developmental time units.

$$GGR(t) = \frac{dW}{dt} = \frac{\lambda\, e^{(-\lambda\,(t-t_0))}\, W_0\left(1+e^{(\lambda\ t_0)}\right)}{\left(1+e^{(-\lambda\,(t-t_0))}\right)^2}$$

Grain growth rate is maximal (GGR_{max}) at the inflexion point, namely t_0.

$$GGR_{max} = GGR(\ t_0) = \frac{\lambda\, W_0\left(1+e^{(\lambda\, t_0)}\right)}{4}$$

with time and model parameters expressed either with time or developmental time units.

Grain filling duration

Grain filling duration (t_f) was considered as the duration between anthesis and the time at which the grain reaches 95% of its final weight. Eq.5 becomes

$$W(t_f) = \frac{W_0\left(1+e^{(\lambda\, t_0)}\right)}{1+e^{(-\lambda\,(t_f-t_0))}} = \frac{95\, W_0\left(1+e^{(\lambda\, t_0)}\right)}{100}$$

$$1+e^{(-\lambda\,(t_f-t_0))} = \frac{100}{95}$$

$$-\lambda\,(t_f - t_0) = \ln\left[\frac{5}{95}\right]$$

And finally,

$$t_f = -\frac{1}{\lambda}\ln\left[\frac{5}{95}\right] + t_0$$

Supplementary Information
Figure S1

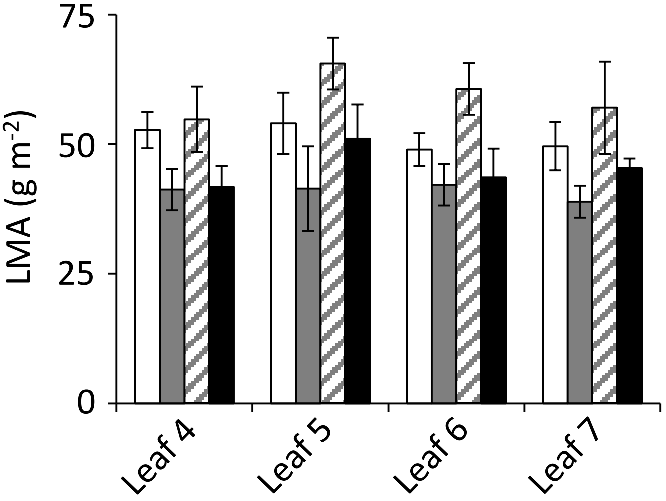

Figure S1. Leaf dry mass per area (LMA) measured at anthesis on 4 leaves for plants grown in different temperature scenarios. (T°$_{day}$ / T°$_{night}$) from the appearance of leaf 4. White bars: 20°C/15°C. Grey bars: 20°C/20°C. Striped bars: 25°C/15°C. Black bars: 25°C/20°C. Bars: average values (n=6); error bars: confidence intervals.

Supplementary Information
Figure S2

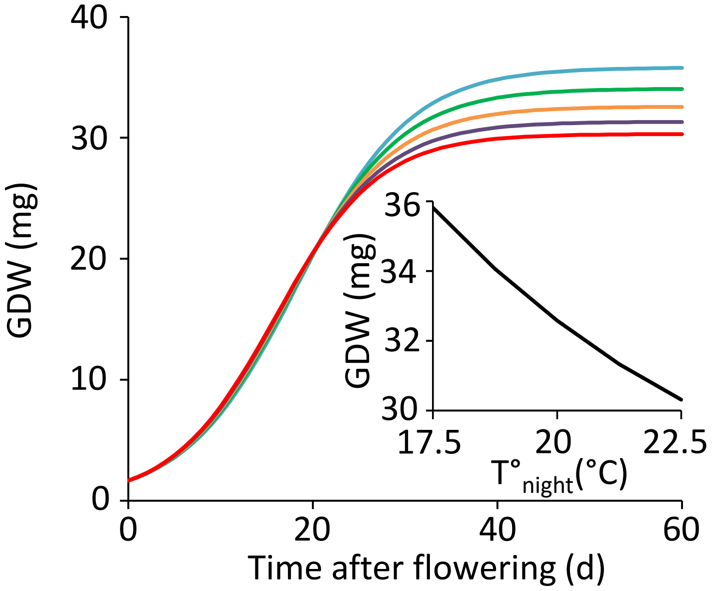

Figure S2. Simulation of the effect of night temperature on time courses of grain dry weight (GDW) and final dry weight (inset). Day temperature is common to all treatment and equal to 25°C. Blue line, $T°_{night}$=17.5°C; green line, $T°_{night}$=18.75°C ; orange line, $T°_{night}$=20°C ; purple line, $T°_{night}$=21.25°C ; red line, $T°_{night}$=22.5°C.

Supplementary Information
Table S1

Table S1. Summary of data coming from the literature.

Article	Source	Growth T° (°C)	Treatment T° (°C)	Beginning (DAA)	End
**Tashiro & Wardlaw (1990)**	Figure 3	21/16	21/16; 30/25	10, 15, 20, 25, 30, 35, 40	10 d after
**Zahedi *et al.* (2003)**	Table 1	20/15	20/15; 30/25	2	maturity
**Warlaw *et al.* (1989a)**	Table 3B	18/13	18/13; 24/19; 30/25	6	maturity
**Warlaw *et al.* (1989b)**	Table 1	18/13	18/13; 30/25	6	maturity
**Warlaw *et al.* (2002)**	Figure 1	18/13	18/13; 21/16; 24/19; 27/22; 30/25	6	maturity
**Zhao *et al*. (2007)**	Figure 7	fluctuating	26/14 and 24/16	7	maturity
**Al-Khatib & Paulsen (1984)**	Table 2	25/15	25/15; 30/20	10	maturity

DAA: Day after anthesis. Only data coming from experiments carried out in controlled environment and with temperatures within the range tested in this paper (10 to 30 °C) were collected. When several cultivars were analyzed, only the average values were considered. For data coming from Tashiro and Wardlaw (1990), only the data for stresses applied after 5 days after anthesis were analyzed because the observed reductions in grain weight for the earliest stresses were mainly due to a decrease in seed number per spike (Fig.1 in the original paper).

Supplementary Information
Table S2

Table S2. Phenotypic data measured in experiment 2.

Variable	$T°_{day}$ / $T°_{night}$: 20°C /15°C	20°C /20°C	25°C /15°C	25°C /20°C
**P_N (mol m^{-2} d^{-1})**	0.700 ± 0.005 (a)	0.721 ± 0.015 (a)	0.839 ± 0.014 (b)	0.852 ± 0.015 (b)
**R (mol m^{-2} d^{-1})**	0.121 ± 0.003 (b)	0.132 ± 0.002 (a)	0.129 ± 0.004 (ab)	0.136 ± 0.001 (a)
**LER (mm d^{-1})**	36.6 ± 1.9 (a)	41.1 ± 3.4 (b)	42.5 ± 4.1 (bc)	46.9 ± 3.1 (c)
**A_N (mol m^{-2} d^{-1})**	0.579 ± 0.012 (a)	0.588 ± 0.018 (a)	0.709 ± 0.015 (b)	0.716 ± 0.016 (b)
**$A_{N.20°C}$ (mol m^{-2} $d_{20°C}^{-1}$)**	0.650 ± 0.010 (b)	0.588 ± 0.018 (a)	0.686 ± 0.015 (c)	0.628 ± 0.018 (b)
**Area (mm^{-2})**	654 ± 57 (a)	718 ± 118 (a)	666 ± 141 (a)	691 ± 122 (a)
**Length (mm)**	129 ± 8 (a)	140 ± 20 (a)	129 ± 20 (a)	135 ± 17 (a)
**Biomass (mg)**	34.2 ± 2.5 (a)	30.7 ± 5.9 (a)	39.5 ± 11 (a)	31.4 ± 5.9 (a)
**LMA (g m^{-2})**	51.6 ± 2.5 (b)	41.4 ± 3.8 (a)	60.0 ± 4.1 (c)	45.4 ± 2.9 (a)

P_N: daily net photosynthesis. R: daily night respiration. LER: elongation rate. A_N, $A_{N.20°C}$: net CO2 assimilation per day or $d_{20°C}$. LMA: Leaf dry mass per area. Data of leaf area, leaf length, leaf biomass and LMA are average values for leaf 4, 5, 6 and 7. Error: confidence interval (n=6). Data associated with the same letter indicate that there were no significant differences in a pairwise t-test.
